# Characterization of Bacterial Communities and Their Antibiotic Resistance Profiles in Wastewaters Obtained from Pharmaceutical Facilities in Lagos and Ogun States, Nigeria

**DOI:** 10.3390/ijerph15071365

**Published:** 2018-06-29

**Authors:** Amarachukwu Obayiuwana, Adeniyi Ogunjobi, Min Yang, Mark Ibekwe

**Affiliations:** 1 Department of Biological Sciences, Augustine University Ilara, Epe 106101, Lagos State, Nigeria; 2 Department of Microbiology, University of Ibadan, Ibadan 200284, Oyo State, Nigeria; ogunjobi@hotmail.com; 3 State Key Laboratory of Environmental Aquatic Chemistry, Research Center for Eco-Environmental Sciences, Chinese Academy of Sciences, Beijing 100085, China; yangmin@rcees.ac.cn; 4 U.S. Salinity Laboratory, USDA-ARS, 450 West Big Springs Road, Riverside, CA 92507, USA; mark.ibekwe@ARS.USDA.GOV

**Keywords:** pharmaceutical wastewater, antibiotics-resistant bacteria (ARB), antibiotic-resistance genes (ARG), sulfonamide resistance genes, mobile genetic elements

## Abstract

In Nigeria, pharmaceutical wastewaters are routinely disseminated in river waters; this could be associated with public health risk to humans and animals. In this study, we characterized antibiotic resistant bacteria (ARB) and their antibiotic resistance profile as well as screening for *sul*1 and *sul*2 genes in pharmaceutical wastewater effluents. Bacterial composition of the wastewater sources was isolated on non-selective media and characterized by the polymerase chain reaction (PCR) amplification of the 16S rRNA genes, with subsequent grouping using restriction fragment length polymorphism (RFLP) and sequencing. The antibiotics sensitivity profiles were investigated using the standard disk diffusion plate method and the minimum inhibitory concentrations (MICs) of selected antibiotics on the bacterial isolates. A total of 254 bacterial strains were isolated, and majority of the isolates were identified as *Acinetobacter* sp., *Klebsiella pneumonia*, *Proteus mirabilis*, *Enterobacter* sp. and *Bacillus* sp. A total of 218 (85.8%) of the bacterial isolates were multidrug resistant. High MICs values were observed for all antibiotics used in the study. The result showed that 31.7%, 21.7% and 43.3% of the bacterial isolates harbored *sul*1, *sul*2, and *Intl*1 genes, respectively. Pharmaceuticals wastewaters are potential reservoirs of ARBs which may harbor resistance genes with possible risk to public health.

## 1. Introduction 

Globally, the development and widespread of resistance to antimicrobial in bacteria [1,2,3] is a major challenge in drug therapy [4,5] in humans and animals. Despite concerted effort to combat this evolving trend by drug combination therapy [6] and other innovative strategies, multidrug resistance (MDR) among bacterial pathogens have posed serious threats to clinical therapy [7,8]. The wide use of antibiotics in humans and animal husbandry has facilitated the increasing spread of MDR. This is particularly worrisome when it is inappropriately used as a result of its availability over the counter without prescription as practiced in most developing countries/regions. This practice and many more have made hospital, municipal, and agricultural sewages major sources of antibiotic residues within the environment [3,9,10].

Until recently, the focus of antibiotic resistance has been on these sources, particularly the clinical sources of MDR development, according to a recent report, contain only a small proportion of the antibiotics resistant determinants found [11]. Many other reports show that treated antibiotic production wastewaters from wastewater treatment plant (WWTPs) contain much higher concentrations of antibiotic residues than other aquatic environments which have been attributed to development of MDR [8,12,13]. It is well known, however, that bacteriophage and virus DNA sequences are very common in influent waste water [14]. In a recent study, diversity of bacteriophage and virus DNA sequences was markedly reduced in effluent water compared to influent [15]. The authors noted that their results of viral DNA analyses obtained in the study were in agreement with other metagenomic studies, showing greater occurrence of bacteriophages compared to human virus sequences in wastewater [16]. 

Several reports have attempted to correlate high concentrations of antibiotics within sewage treatment plants (STPs) with increased levels of resistance to antibiotics by bacteria [17] without clarity in outcomes. Environmental bacteria in STPs have been demonstrated in many studies as carriers of antibiotics resistance determinants [18,19,20,21] and potential sources of novel resistance genes in clinical pathogens [22,23]. In addition, in agricultural practice, the use of treated wastewater effluent as alternative source of irrigation water may introduce active antibiotic resistant pathogens to the soil [24,25] which may pose health risk to humans that come in contact with them. Owing to the limited availability of clear evidence showing the evolution of resistance and the spread of antibiotics resistance genes (ARGs) in WWTPs [11], there is need to extend antibiotic resistance studies to WWTPs beyond clinical studies. 

The dihydropteroate synthase (DHPS) genes *sul*1 and *sul*2 have been detected in bacterial isolates from water and other aquatic environments [26,27], and even from rivers and seawater without evidence of being polluted [28,29,30]. The *sul*1 gene, as a part of class 1 integron, can be disseminated and transferred horizontally within and between species in wastewater [31], river water [32], and seawater [33]. It has been found linked to other resistance genes in class 1 integrons and on large conjugative plasmids [34] while *sul*2 is usually located on small nonconjugative plasmids [35], large transmissible multiresistance plasmids [36], or through insertion element common region (ISCR2) element [37]. Studies on the presence of the dihydropteroate synthase (DHPS) genes and the integron reveal the possibility of dissemination of these genes within water sources.

In this study, we investigated the antibiotic resistance profile of bacterial isolates obtained from wastewater samples collected from fourteen pharmaceutical facilities in Lagos and Ogun States Southwestern Nigeria over a twenty-six-month period. The selected pharmaceutical facilities are key players in antibiotic production at the secondary and tertiary stage of production in these regions. In Nigeria, most pharmaceutical industries produce various antibiotics and other drug types in a single production plant. In most cases, they lack wastewater treatment before the effluents are released into the environment and other bodies of water [38]. The best practice is to hold the wastewater over a period; in some cases, the wastewater is diluted. The untreated wastewater is either deposited underground or discharged directly into nearby natural water bodies [38]. We also investigated a wastewater treatment plant situated in an industrial Estate which receives wastewater from pharmaceutical facilities production plants and other production factories within the region. In addition, household sewages from residential quarters were collected within the treatment plant. We further studied the river water samples obtained from the site where the effluent is discharged at the end of treatment. Conventional wastewater treatment methods were employed within the system.

## 2. Materials and Methods 

### 2.1. Study Sites and Sampling

The pharmaceutical wastewater samples were obtained directly from 14 pharmaceutical companies in Agbara, Sango-Ota, Ikeja, Oshodi and Isolo towns of Ogun and Lagos States, Southwestern Nigeria (Figure S1) over a twenty-six month period, between February 2011 and April 2013. In addition, municipal wastewater samples were collected from a central wastewater treatment plant located in Agbara Industrial Estate of Ogun State (Figure S2, Plate S1). Otoawori sand beach river water (upstream and downstream) was sampled as the discharge point for the final effluents. A total of 20 samples from the 14 sites were collected from Agbara, Sango-Ota, Ikeja, Oshodi and Isolo towns of Ogun and Lagos States, including the WTP and RW samples (Table S1). The samples were collected in duplicates, and were used as composite samples. Samples were taken aseptically in 2-L brown glass bottles, kept at 4 °C in the dark and bacterial isolation was carried out within 24 h of collection. 

### 2.2. Bacterial Isolation and Counts

For isolation of bacteria, wastewater and river water samples were serially diluted in 10-fold physiological saline and 1.0 mL aliquots of appropriate dilutions (10^−2^–10^−6^) were inoculated and plated on non-selective agar media, tryptone soya agar (TSA) and plate count agar (PCA) (Oxoid Ltd., Basingstoke, Hampshire, UK). Duplicate plates were incubated under aerobic condition at 35 °C for up to 48 h. Bacterial counts were taken every 24 h of incubation. Morphologically distinct colonies were subcultured onto fresh plates of nutrient Agar (NA) (Oxoid Ltd., Basingstoke, Hampshire, UK). Up to eight colonies with different morphologies were taken from each plate. Isolates were restreaked up to three times and purity was verified by Grams reaction and microscopy. Pure colonies of each isolated bacteria strain were stored on NA slants at 4 °C, and for prolonged storage, at −20 °C in tryptic soy broth (TSB) containing 15% glycerol. 

### 2.3. Bacterial Identification

Genomic DNA was extracted from all the bacterial isolates by the use of TIANamp bacteria DNA kit (TIANGEN Biotech Co., Beijing, China), and also by the boiling and thawing method [8]. The 16S rRNA genes from pure cultures were ampli**fi**ed using bacterial universal primers 27F and 1492R (Table 1) [39] for the target of the conserved region of the 16S rRNA of the bacteria. The standard 50 µL PCR mixture (Takara, Dalian, China) was used. The composition was 1× PCR buffer containing 1.5 mM MgCl_2_, 200 mM of each deoxynucleoside, triphosphate (dNTP), 10 pmol of each primer, 1.25 U of TaKaRa© rTaq polymerase, and 1 µL of DNA template. Polymerase chain reaction conditions consisted of initial denaturation at 95 °C for 5 min, followed by 30 cycles of 95 °C for 1 min, annealing temperature at 55 °C for 1 min, extension at 72 °C for 1 min 30 s, and completed with a final extension at 72 °C for 10 min. Sterile water was used as the negative control. The amplification of the 16S rRNA genes was confirmed by electrophoresis in 1.2% (*w*/*v*) agarose gel, amplified products were purified with the Qiaquick PCR cleanup kit (Qiagen, Chatsworth, CA, USA) following the manufacturer’s instructions.

The ampli**fi**ed products were grouped according to the analysis of *Hae*III (Takara, Dalian, China) restriction fragment length polymorphism (RFLP) patterns. The reaction mixture contains 1 µL of *Hae*III enzyme, 2 µL 10 × M buffer, 20 µL of sterilizes distilled water, and 1 µg of purified DNA substrate. The mixture was incubated at 37 °C for 6 h. The product was analyzed on 2% (*w*/*v*) agarose gel. The RFLP patterns were analyzed using BioNumerics version 6.01 (Applied Maths, SintMartens-Latem, Belgium) [8]. For each RFLP pattern, one or two amplified 16S rRNA gene representative products were sequenced (ABI 3730 capillary sequencer [Applied Biosystems]) and classified by construction of phylogenetic trees using the neighbor-joining algorithm with Ribosomal Database Project II release 9.49 and the GenBank database using the BLAST program [40,41].

### 2.4. Antibiotic Susceptibility Testing

The susceptibility of bacterial isolates from the wastewater and surface water to antibiotics was tested on Mueller Hinton agar using the Kirby–Bauer disc diffusion method [42]. Antibiotic sensitivity discs (Abtek) employed contained augmentin (30 µg), ofloxacin (5 µg), gentamicin (10 µg), nalidixic acid (30 µg), nitrofurantoin (200 µg), cotrimoxazole (25 µg), amoxycillin (25 µg), and tetracycline (25 µg). According to standard procedures, the sensitivity discs were carefully layered on each plate and the plates were incubated overnight at 37 °C, after which zones of growth inhibition around each disc were measured and interpreted by the zone breakpoint standards of the Clinical and Laboratory Standards Institute [43]. 

The MICs of antibiotics for the bacterial isolates were determined by a standard two-fold serial broth microdilution method using Mueller–Hinton broth according to the NLCC Standards guidelines [43], with antibiotic concentrations ranging from 0.5 to 1024 µg/mL. The following 12 antibiotics representing six classes were tested: β-Lactams including Ampicillin (AMP) and Amoxicillin (AMO); Aminoglycosides including Streptomycin (STR) and Kanamycin (KAN); Macrolides including Erythromycin (ERY), Spiramycin (SPI) and Chloramphenicol (CHL); tetracyclines including Tetracycline (TET) and Oxytetracycline (OXY); quinolones including Nalidixic Acid (NAL); and Sulfonamides including Sulfamethoxazole (SUL) and Trimethoprim (TRI). All antibiotics and chemicals, except Kanamycin, were obtained from Sigma-Aldrich Chemie Gmbh, Buchs SG, Switzerland. All antibiotic solutions were prepared according to manufacturer’s instructions. *Escherichia coli* ATCC 25922 and ATCC 35218 and *Pseudomonas aeruginosa* ATCC 27853 were used as controls. The resistance prevalence for an antibiotic in a bacterial population was calculated as the ratio of the number of strains resistant to the particular antibiotic versus the total number of strains in the population [8].

### 2.5. PCR Detection of Sulfonamide Resistance Genes and Class I Integrons

The bacterial isolates from all the water samples were screened for the presence of 2 sulfonamide resistance genes (*sul*1 and *sul*2) and also for class 1 integrons (*intl*1). This was determined by PCR, using the standard PCR mixture (50 µL) as described above. Bacterial DNA was used as template. The PCR primers and conditions for ampli**fi**cation of *sul* genes and are listed in Table 1. All PCR experiments included positive controls (genomic DNA carrying *sul* genes or class I integrons) and a negative control (PCR mixture without DNA template). Amplified products were separated by 2% (wt/vol) agarose gel electrophoresis and visualized by ethidium bromide staining. DNA molecular weight marker pBR 328 (Roth, Germany) was used as a standard DNA ladder.

### 2.6. Statistical Analysis 

Statistical analysis was performed using excel and SPSS 16.0. The correlation analysis was used to calculate the Pearson’s bivariate correlation and *p*-values.

### 2.7. Nucleotide Sequence Accession Numbers 

The 16S rRNA gene sequences of bacterial isolates in this study were deposited in the GenBank database with accession No. MH396719-MH396771.

## 3. Results

### 3.1. Total Bacterial Counts

The samples collected at the three different sampling times showed high number of bacteria (CFU/mL) for each of the samples. The results were categorized into Wastewater (WW) samples, Wastewater Treatment Plant (WTP) samples, and River Water (RW) samples. As shown in Table 2, at the end of the 48 h of bacterial incubation on TPC and PCA plates, bacterial counts (CFU/mL) of the WW, WTP, and RW samples showed a range of 1.2 × 10^4^–2.2 ×10^8^, 3.6 × 10^5^–2.6 × 10^6^ and 0.6–2.2 × 10^5^ CFU/mL, respectively (Supplementary Materials, Table S2).

### 3.2. Composition of Bacterial Isolates

**Figure 1:**
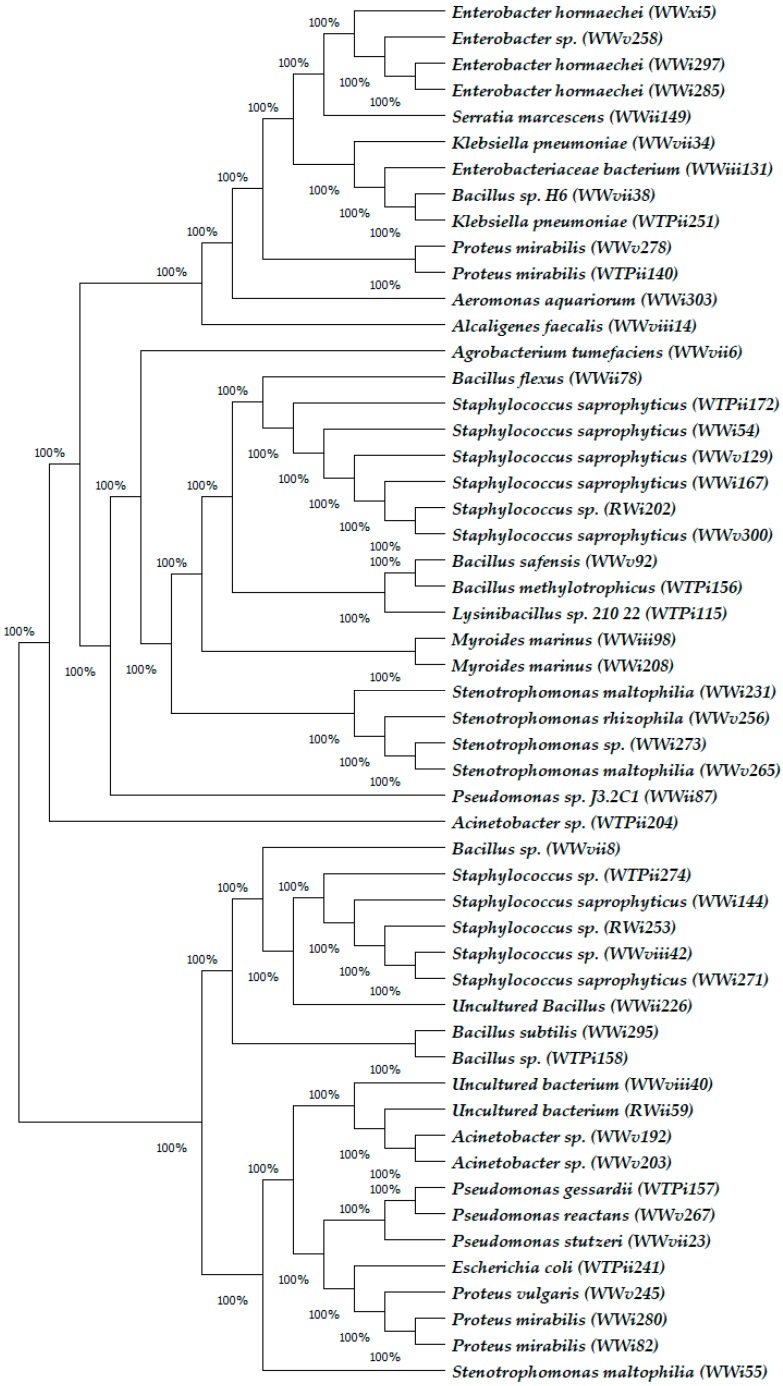
Neighbor-joining Phylogenetic tree of Bacterial isolates obtained from WW, WTP and RW. Parenthesis indicates species code of bacterial isolate, indicating isolate sources.

In total, 254 bacterial isolates were obtained from the water samples on non-selective media: 183 from WW samples, 33 from WTP samples and 38 from the RW samples (Table 2). Phylogenetic groups were determined for each bacterial sequence obtained from this study (Figure 1) with high similarities (99–100%) to known species based of BLAST (National Centre for Biotechnological Information (NCBI). The result shows that the environmental isolates in this study belong to at least 16 different genera and unculturable group (Table 2). The 16 genera are *Acinetobacter*, *Aeromonas*, *Agrobacterium*, *Alcaligenes*, *Bacillus*, *Enterobacter*, *Enterobacteriaceae*, *Escherichia*, *Klebsiella*, *Lysinibacillus*, *Myroides*, *Proteus*, *Pseudomonas*, *Serratia*, *Staphylococcus*, and *Stenotrophomonas*. The majority of the organisms obtained belong to the bacterial division Gammaproteobacteria (66.6%). The other bacterial isolates belong to the bacterial division Alphaproteobacteria (0.8%), Betaproteobacteria (0.4%), Firmicutes (25.2%), Bacteroidetes (3.9%) and the unculturable group (3.1%). The most prevalent species were the *Proteus mirabilis* isolates, followed by *Acinetobacter* sp. and *Enterobacter* sp. Figure 1 shows the Neighbor-joining Phylogenetic tree of the bacterial isolates from the WW, WTP and RW.

### 3.3. Antibiotics Resistance Prevalence and MDR

The result of the antimicrobial susceptibility testing for the 254 bacterial isolates using Kirby–Bauer disc diffusion method showed 54 distinct phenotypic patterns of resistance. Percentage resistance to the test antibiotics varied between 5.3% for Gentamicin among isolates from RW samples to 98.3% in Amoxycillin for isolates obtained from WW samples (Table 3). Table 3 shows that 243 bacterial isolates were resistant to Augmentin while 54 showed resistance to Ofloxacin. Overall, 218 bacterial isolates were multidrug resistance to the test antibiotics, which represents 85.8% of the total tested bacterial isolates. 

The minimum inhibitory concentration (MIC) result of antibiotics shows that the resistance prevalence for almost all antibiotics tested in this study was high in all the bacterial isolates (Table 4). The resistance prevalence to kanamycin was the lowest (54.7%) amongst the antibiotics tested. All the bacterial isolates showed MIC for sulfonamide greater than or equal to 1024 mg/L, indicating a high resistance by all the test isolates to sulfonamide. *Acinetobacter* sp. Obtained from the wastewater treatment plant showed the highest resistance to the test antibiotics with highest MIC (≥1024 mg/L) in all the tested antimicrobial except for Streptomycin Sulfate, Tetracycline and Erythromycin that showed MICs ≥ 512 mg/L.

As shown in Table 4, the antibiotic levels of the bacterial communities in all three water samples were reflected by the MIC50s and MIC90s, which represent MICs required for the inhibition of 50% and 90% of bacterial strains, respectively. There were no significant differences in the MIC50 and MIC90 values for all 12 antibiotics tested within the bacterial communities of the samples (Wilcoxon matched-pair test, both *p* values were >0.1). MIC50 and MIC90 values of tetracycline were lowest amongst the tested antibiotics, while the values for ampicillin, amoxicillin, trimethoprim, chloramphenicol and sulfonamides are the highest with 1024 mg/L for MIC50 and MIC90 values, respectively. Almost all the bacterial isolates from all sources (more than 96%) exhibited MDR. There was 100% resistance prevalence in ampicillin, amoxicillin, trimethoprim, chloramphenicol and sulfonamides antibiotics. Kanamycin has the lowest resistance prevalence of the 12 antibiotics tested.

### 3.4. Sulfonamide Resistance Genes

Sulfonamide resistance genes *sul*1 and *sul*2 were detected in 31.7% and 21.7% of the bacterial isolates, respectively. About 15% of the bacterial isolates from the water samples harbored both *sul*1 and *sul*2 resistance genes. *Bacillus methylotrophicus*, *Acinetobacter* sp., *Klebsiella pneumonia*, *Enterobacter hormaechei*, *Serratia marcescens* and *Staphylococcus saprophyticus* are some of the bacterial isolates that harbored both *sul*1 and *sul*2 antibiotic resistance genes. These *sul*-positive isolates were generally not susceptible to Sulfonamides. 

The mobile genetic elements, class I (*Intl*1) and class II (*Intl*2) integrons were screened for in the genomic DNA samples. The *intl*1 genes were identified in 43.3% of the bacterial isolates and *Intl*2 was not detected in any of the bacterial cells screened (Table 5). *Staphylococcus saprophyticus*, *Aeromonas aquariorum*, *Enterobacteriaceae bacterium* and *Klebsiella pneumonia* are bacteria isolates that harbored *intl*1, *sul*1 and *sul*2 genes. Other groups of resistance genes were found in the sequenced integrons, they include the aminoglycoside resistance genes, which are not discussed in this report.

## 4. Discussion

The results obtained in this study indicate that the pharmaceutical wastewater environment contains a large community of bacteria (Table 2). High bacteria counts were observed in the WW, WTP and RW samples (Supplementary Materials, Table S2). Two pharmaceutical facilities (WWi2 and WWv) showed the highest bacteria count in the study. The wastewater samples from the wastewater treatment plant (WTPi and WTPii) also have high bacterial counts. The treated wastewater WTPii have more bacteria counts than the untreated wastewater WTPi. The river water samples RWi and RWii have almost the same bacteria counts, but the upstream water RWi has more count compared to the downstream RWii sample. This is expected because the upstream source is closer to the WTP discharge point. 

The result of the analysis of the 16S rRNA genes in the bacterial isolates from wastewater and surface water samples in this study showed that these environments harbor very viable bacteria population. The Gammaproteobacteria was the most frequent bacteria isolates in this study, which could be because of their favored growth in nutrient-rich culture media. This observation agrees with the work of Li et al. [8] where the majority of the 341 bacterial isolates obtained from an oxytetracycline WTP and river water were Gammaproteobacteria. *Proteus mirabilis* and *Acinetobacter* sp. were the most abundant bacteria isolates obtained from this study. In a similar investigation, Guardabassi et al. [46] demonstrated that discharge of wastewater from a pharmaceutical plant was associated with an increase in the prevalence of both single and multiple-antibiotic resistance among *Acinetobacter* species in the sewers. 

The bacterial isolates from the sample groups showed high levels of resistance to all the tested antimicrobials. Among the 254 bacteria isolates, a large number (95.7%) of the bacteria isolates showed high resistance to Augmentin, a penicillin combination of Amoxycillin and Clavulanic Acid, which are classified as beta-lactams. The combination is expected to have very high efficacy against infectious organisms. This antibiotic is a choice therapy in Nigeria in both children and adult. This high resistance to Augmentine agrees with the work of Li et al. [47] which shows that resistance to β-lactams antibiotics was more frequent, with much higher levels, than the other classes of antibiotics tested in isolates of a penicillin production wastewater treatment plant and receiving river. The MIC result shows a universal resistance to sulfonamide antibiotics. This result instigated the investigation of the sulfonamide resistance genes in this study. 

In WW samples, antibiotic resistance phenotypes were very common. Resistance (93.4%) to more than three classes of the tested antibiotic was observed in the WW isolates. In Agbara Ogun State, WWii samples showed multi-drug resistant (MDR) strains in all the bacterial isolates, except for three of the isolates that showed resistant to less than three classes of the tested antibiotics. Samples of WWv showed all bacterial isolates to be MDR. In Sango Ota Ogun State, out of the 32 WWi wastewater samples, seven of the bacterial isolates showed resistant to all the tested eight antibiotics. On the other hand, all isolates are MDR except for one. Most of the MDR organisms showed resistant mostl to 5–7 classes of the tested antibiotics, but at least to four of the classes. In wastewater samples of WWiii in Ikeja Lagos State, all the bacterial isolates encountered are MDR. In addition, in sample WWvii, all the 38 isolates were MDR except for one. All the bacterial isolates obtained from WWx samples in Oshodi Lagos State were all MDR with resistant to at least six classes of the antibiotics tested. With exception of one non-MDR isolate, all bacterial isolates obtained from WWxi were MDR. This is in line with a recent investigation by Li et al. [8] in oxytetracycline waste water treatment plant demonstrated that strong selective pressure was imposed by a high concentration of oxytetracycline which contributed to the proliferation of MDR bacteria strains in the wastewater environment. 

The presence of antibiotics in the report above might be a single factor for the selection of MDR bacterial isolates. Wastewater treatment plants are interfaces between different environments and it has often been reported to have a high level of residual antibiotics. Li et al. [47] investigated the bacterial characteristics of a penicillin production wastewater treatment plant and the receiving river and demonstrated that high resistance prevalence and levels could be induced by long-term penicillin exposure. This present study detected high level of antibiotics (result not presented in this report) in wastewater coming from a production line in a pharmaceutical facility (WWx) in Oshodi Lagos State. This condition might be the reason for selection of only MDR bacterial isolates in this facility. Although it is still difficult to establish clear cause effect relationships, it is widely accepted that chemical pollution contributes to antibiotic resistance dissemination [48,49,50]. 

The WTPi bacteria isolates have six MDR, whereas the WTPii have 17 MDR bacteria isolates out of the total number of 26 isolates. This result again questions the efficacy of the treatment process, though some studies have demonstrated that wastewater treatment processes, operating according to legal recommendation, cannot reduce effectively the levels of antibiotic resistance [19]. The increase in MDR bacteria isolates in WTPii compared to WTPi suggests that the treatment procedure in the WTP might have selected for more MDR strains. This observation may be in line with the report of Manaia [51] that wastewater composition and the treatment process itself may pose selective pressures capable of modulating either the bacterial populations or the antibiotic resistance pool. 

In the river samples, RWi (upstream) and RWii (downstream) have 18 bacterial isolates each. The upstream samples had 10 MDR, whereas the downstream bacteria isolates had 12. It is expected that more MDR will be observed in the upstream compared to the downstream samples. The number of bacteria isolates obtained from these compartments is not enough to draw major inferences in these two segments of the river water samples. The major reason for the analysis of the river water is to screen for the presence of antibiotic resistance genes or other antibiotic resistance determinants within the aquatic confinement. The presence of antibiotic in the river waters might also pose selective pressure on antibiotics resistance determinants. Although this comes in much lower concentration, reviews have it that pharmaceuticals is not only found in wastewaters, but also in surface, ground and drinking waters [52,53,54]. The fact established in this study is that MDR bacteria are found within the river water environment.

Multi resistant bacteria are prevalent in this study and encountered in all the tested isolates. In this study, sulfonamide resistance genes *sul*1 and *sul*2 were detected in 31.7% and 21.7% of the bacterial isolates, respectively. This supports the high resistance to the sulfonamide antibiotics used in the antibiotics susceptibility test in this study. Since they all showed resistance to sulfonamide antibiotics, the question is: Why were sulfonamide resistance genes not found in all the bacterial isolates? There is a high chance that other sulfonamide resistance genes other than the *sul*1 and *sul*2 were responsible for the resistance to sulfonamide recorded, because different types of mechanisms have been found to confer resistance to sulfonamide, mostly based on changes in the *sul* genes and mediation by mobile elements [55]. There is also a possibility that beyond the sulfonamide resistance genes, there could be other factors responsible for conferring resistance of sulfonamide to the bacterial isolates. In addition, the detection of *sul* ARGs does not mean that they are conferring resistance in the host; more sophisticated studies are needed to distinguish between ARG carriage in the host chromosome and ARG which confers resistance to the treatment of pathogens [11].

Integrons, especially class I integrons, commonly contain antibiotic resistance gene cassettes and are closely related to MDR, generally by containing several resistance gene cassettes simultaneously [56,57]. In the result presented above (Table 5), integron 1 (*Intl*1) was detected in 43.3% of the bacterial isolates while *Intl2* was not detected. The presence of *Intl*1 in the isolates indicates a high prevalence within the wastewater and river water medium. The relative abundance of the clinical class 1 integron-integrase gene, *intI*1, is a proxy for anthropogenic pollution amongst many other factors is that they are linked to genes conferring resistance to antibiotics [56]. This situation can pose a high possibility of dissemination of resistance determinants within the water systems from one bacterium to the other and possibly to clinical isolates in some instances.

Studies on antibiotics resistome in different environmental compartments have been carried out in many regions worldwide. However, this is the first report on the attempt to elucidate the antibiotic resistance profile of bacterial community within the pharmaceutical wastewater in the Nigerian environment, under possible range of anthropogenic influence. The overview of our findings suggests that antibiotic resistance status in the Nigerian environment is no different from what is obtainable in other regions. This result can give insight to understanding the emergence and dissemination of novel antibiotics resistance from the natural reservoirs to the clinical environments. 

## 5. Conclusions

Pharmaceutical wastewater and wastewater treatment plants are potential hot spots of selection of antibiotic resistance and dissemination of genetic determinants of antibiotic resistance. Our study clearly shows this by the high level ARBs isolated from the wastewater. The presence of mobile genetic elements *Intl*1 from the environmental sources have the likelihood to promote the dissemination of drug resistance determinants amongst related bacteria species. This and the possible link of these wastewaters to the waterways could imply the transfer of antibiotic resistance to the general populace with possible public health implications. The overall results suggest that our environment is not free from antibiotic resistant bacteria and resistance genes, but may harbor novel resistance genes. This possibility amongst many others hint on the importance of initiating more vigorous surveillance programs to monitor the wastewater management of pharmaceutical outlets, to keep abreast the environmental integrity of our aquatic ecosystem.

## Figures and Tables

**Table 1:** Primers and conditions used to amplify 16S rRNA genes, sul genes, class 1 and 2 integrons by PCR technique.

Target Gene	Sequence (5′-3′)	Amplicon Size (bp)	Annealing Temp. (°C)	Reference
27 F	AGAGTTTGATCCTGGCTCAG	1503	55	[39]
1492 R	TACGGYTACCTTGTTACGACTT			
*Sul*I, F	ATCGCAATAGTTGGCGAAGT	798	55	[44]
*Sul*I, R	GCAAGGCGGAAACCCGCGCC			
*Sul*II, F	GCGCTCAAGGCAGATGGCATT	284	70	[44]
*Sul*II, R	GCGTTTGATACCGGCACCCGT			
*intI*1, F	CCTCCCGCACGATGATC	280	55	[45]
*intI*1, R	TCCACGCATCGTCAGGC			
*intI*2, F	TTATTGCTGGGATTAGGC	233	50	[45]
*intI*2, R	ACGGCTACCCTCTGTTATC			

F—Forward Primer; R—Reverse Primer.

**Table 2:** Frequency Distribution of Bacterial Strains isolated from wastewater, wastewater treatment plant and river water.

Genus or Species	No of Isolates from			Total Number of Isolates
	^I^WW	WTP	RW	
*Acinetobacter* sp.	25	1	5	31
*Aeromonas aquariorum*	3	-	-	3
*Agrobacterium tumefaciens*	3	2	-	5
*Alcaligenes faecalis*	1	-	-	1
*Bacillus flexus*	5	2	1	8
*Bacillus methylotrophicus*	3	1	1	5
*Bacillus safensis*	5	-	1	6
*Bacillus subtilis*	5	2	1	8
*Bacillus* sp.	15	1	2	18
*Enterobacter hormaechei*	6	1	1	8
*Enterobacter* sp.	20	1	3	24
*Enterobacteriaceae bacterium*	1	-	2	3
*Escherichia coli*	2	1	-	3
*Klebsiella pneumoniae*	14	2	2	18
*Lysinibacillus* sp. *210_22*	2	1	-	3
*Myroides marinus*	6	1	2	9
*Proteus mirabilis*	24	5	6	35
*Proteus vulgaris*	5	2	1	8
*Pseudomonas gessardii*	1	1	-	2
*Pseudomonas reactans*	2	-	-	2
*Pseudomonas* sp.	6	3	4	13
*Pseudomonas stutzeri*	1	-	-	1
*Serratia marcescens*	3	1	-	4
*Staphylococcus saprophyticus*	5	2	2	9
*Staphylococcus* sp.	6	1	2	9
*Stenotrophomonas maltophilia*	4	1	1	6
*Stenotrophomonas rhizophila*	2	-	-	2
*Stenotrophomonas* sp.	2	-	-	2
Uncultured bacterium	6	1	1	8
TOTAL	**183**	**33**	**38**	**254**

^I^WW, wastewater effluent; WTP, wastewater treatment plant; RW, river water. - denotes that organism not isolated from the sample.

**Table 3:** Sensitivity of the bacteria isolated from different wastewaters (WW and WTP) and river water (RW) to selected antibiotics using the Kirby–Bauer disc diffusion method.

Class of Antibiotics	Group	^I^WW	WTP	RW	Total
Penicillin/Clavulanic acid	Augmentin	178 (97.3)	30 (90.9)	35 (92.1)	243 (95.7)
Quinolones	Ofloxacin	47 (25.7)	2 (6.1)	5 (13.2)	54 (21.3)
	Nalidixic Acid	82 (44.8)	5 (15.2)	4 (10.5)	91 (35.8)
Aminoglycosides	Gentamicin	46 (25.1)	11 (33.3)	2 (5.3)	59 (23.2)
Nitrofurans	Nitrofurantoin	89 (48.6)	15 (45.5)	21 (55.3)	125 (49.2)
Sulfonamides	Cotrimoxazole	158 (86.3)	14 (42.4)	16 (42.1)	188 (74.0)
Penicillins	Amoxycillin	180 (98.3)	23 (69.7)	31 (81.6)	234 (92.1)
Tetracyclines	Tetracycline	127 (69.4)	15 (45.5)	5 (13.2)	147 (57.9)
MDR	3 classes and above	171 (93.4)	22 (66.7)	25 (65.8)	218 (85.8)
NMDR	Less than 3 classes	12 (6.6)	11 (33.3)	13 (34.2)	36 (14.2)
**Total Isolates**	-	**183**	**33**	**38**	**254**

WW, wastewater effluent; WTP, wastewater treatment plant; RW, river water.

**Table 4:** Minimum inhibitory concentration (MIC) of 12 antibiotics against bacteria isolates obtained from WW, WTP and RW.

Activity against the Isolates				
Antibiotics	Resistance Prevalence (%)	MIC (mg L^−1^)		
		Range	50%	90%
Ampicillin (AMP)	100.0	64 to ≥1024	1024	1024
Amoxicillin (AMO)	100.0	1 to ≥1024	1024	1024
Streptomycin Sulfate (STR)	92.3	1 to ≥1024	128	512
Trimethoprim (TRI)	98.0	8 to ≥1024	1024	1024
Chloramphenicol (CHL)	100.0	256 to ≥1024	1024	1024
Sulfonamide (SUL)	100.0	512 to ≥1024	1024	1024
Tetracycline (TET)	90.6	2 to ≥512	128	256
Oxytetracycline (OXY)	90.6	1 to ≥1024	256	512
Nalidixic Acid (NAL)	73.6	1 to ≥1024	512	1024
Erythromycin (ERY)	92.5	4 to ≥1024	128	512
Spiramycin (SPIRA)	90.6	2 to ≥1024	512	1024
Kanamycin (KAN)	54.7	1 to ≥1024	128	1024

WW, wastewater effluent; WTP, wastewater treatment plant; RW, river water; 50%, MIC50; 90%, MIC90. The MICs for each antibiotic for all tested isolates in WW, WTP, and RW, which represent MICs required for the inhibition of 50% and 90% of bacterial strains respectively.

**Table 5:** Prevalence of Sulfonamide Resistance Genes and Mobile Genetic Elements in Bacterial Isolates Samples.

Genes Class	Resistance Genes	Bacterial Isolates (%)
Sulfonamide genes	*sul*1	31.7
	*sul2*	21.7
Mobile genetic elements	*Intl*1	43.3
	*Intl*2	0

## Supplementary Materials

The following are available online at http://www.mdpi.com/1660-4601/15/7/1365/s1, Table S1: Description of sampled sites; Table S2: Colony Forming Unit per mL (CFU/mL) for the Environmental Samples under study on Total Plate Count Agar. Figure S1: Map of Nigeria highlighting the study areas: Ogun and Lagos States; Figure S2: A section of the map of Nigeria showing Agbara Industrial Area in Ogun State; Plate S1: Cross-section of the wastewater treatment Plant located in Agbara Industrial Estate, in Ogun State, Nigeria.
