# Supplementary material for: Characterization of Bacterial Communities and Their Antibiotic Resistance Profiles in Wastewaters Obtained from Pharmaceutical Facilities in Lagos and Ogun States, Nigeria
Source: Int J Environ Res Public Health. 2018 Jun 29;15(7):1365. doi: 10.3390/ijerph15071365 (PMC6069043; doi:10.3390/ijerph15071365)
Supplement: Supplementary file 1 [file ijerph-15-01365-s001.pdf]

Supplementary Materials

Table S1. Description of sampled sites.

Area	Sample codes	Site Location	Sample	Sample description
Agbara Ogun State	WTPi	6^0 30' N 3^0 5' E	Untreated wastewater	Central untreated wastewater holding tank
	WTPii		Treated water	Treatment tank
	RWi	6^0 29' N 3^0 6' E	River water	Upstream of receiving water
	RWii	6^0 29' N 3^0 6' E	River water	Downstream of receiving water
	WWii1	6^0 30' N 3^0 4' E	Wastewater	Wastewater channel
	WWii2	6^0 30' N 3^0 4' E	Wastewater	Wastewater holding tank
	WWiv	6^0 30' N 3^0 6' E	Wastewater	Wastewater outlet
	WWv	6^0 30' N 3^0 4' E	Wastewater	Stored wastewater
Sango Ota Ogun State	WWi1	6^0 30' N 3^0 5' E	Wastewater	Holding tank
	WWi2	6^0 30' N 3^0 5' E	Wastewater	Discharge point
	WWvi	6^0 42' N 3^0 13' E	Wastewater	Outlet to the public drainage
Ikeja-Lagos State	WWiii	7^0 39' N 5^0 3' E	Wastewater	Holding thank
	WWvii	9^0 46' N 5^0 56' E	Wastewater	Discharge channel
	WWviii	6^0 10' N 5^0 18' E	Wastewater	Holding tank
	WWix	-	Wastewater	Wastewater channel
Oshodi-Lagos State	WWx	-	Wastewater	Holding tank wastewater
Isolo-Lagos State	WWxi	-	Wastewater	Holding tank
	WWxii	-	Wastewater	Discharge point
	WWxiii	-	Wastewater	Holding tank
	WWxiv	-	Wastewater	Holding tank

International Journal of
Environmental Research and Public Health

MDPI

Table S2. Colony Forming Unit per mL (CFU/mL) for the Environmental Samples under study on Plate Count Agar.

S/N	Sample Codes [a]	CFU/mL
1	**WWi1**	3.8×10^{5}
2	**WWi2**	2.2×10^{8}
3	**WWii1**	4.8×10^{5}
4	**WWii2**	0.3×10^{7}
5	**WWiii**	4.5×10^{5}
6	**WWiv**	3.7×10^{5}
7	**WWv**	2.2×10^{8}
8	**WWviii**	6.6×10^{5}
9	**WWix**	1.2×10^{4}
10	**WWxii**	0.1×10^{7}
11	**WTPi**	3.6×10^{4}
12	**WTPii**	2.6×10^{6}
13	**RWi**	2.2×10^{5}
14	**RWii**	0.6×10^{5}

[a] WW denotes Wastewaters from individual pharmaceutical facilities; WTP wastewater from the Wastewater Treatment Plant; RW, River Water.

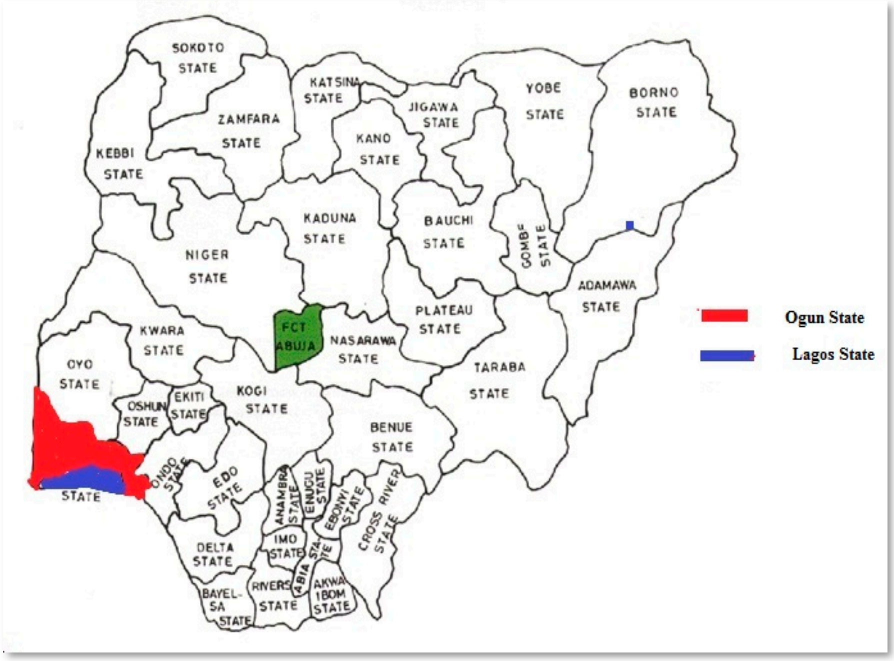

Figure S1. Map of Nigeria highlighting the study areas: Ogun and Lagos States.

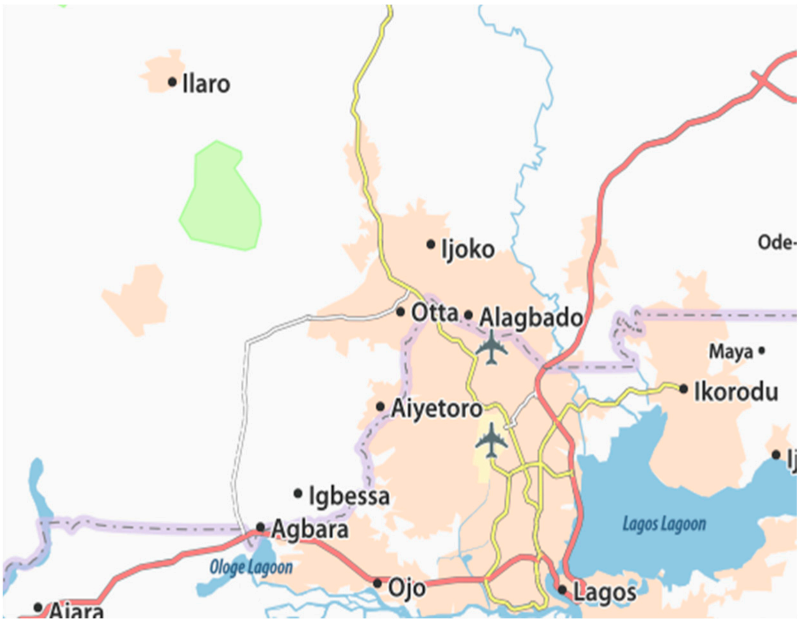

Figure S2. A section of the map of Nigeria showing Agbara Industrial Area in Ogun State.

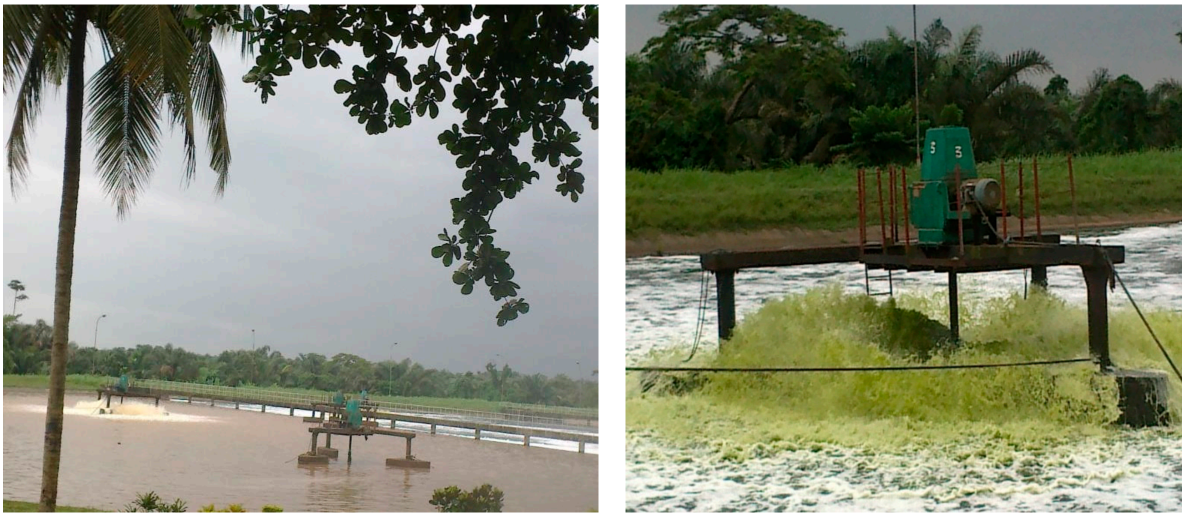

Plate S1. Cross-section of the wastewater treatment Plant located in Agbara Industrial Estate, in Ogun State, Nigeria.

© 2018 by the authors. Submitted for possible open access publication under the terms and conditions of the Creative Commons Attribution (CC BY) license (http://creativecommons.org/licenses/by/4.0/).
